# Genotype List String: a grammar for describing HLA and KIR genotyping results in a text string

**DOI:** 10.1111/tan.12150

**Published:** 2013-07-12

**Authors:** R P Milius, S J Mack, J A Hollenbach, J Pollack, M L Heuer, L Gragert, S Spellman, L A Guethlein, E A Trachtenberg, S Cooley, W Bochtler, C R Mueller, J Robinson, S G E Marsh, M Maiers

**Affiliations:** 1 Department of Bioinformatics, National Marrow Donor ProgramMinneapolis, MN, USA; 2 Center for Genetics, Children's Hospital Oakland Research InstituteOakland, CA, USA; 3 Center for International Blood and Marrow Transplant ResearchMinneapolis, MN, USA; 4 School of Medicine, Stanford UniversityStanford, CA, USA; 5 Masonic Cancer Center, University of MinnesotaMinneapolis, MN, USA; 6 Zentrales Knochenmarkspender-Register für Deutschland (ZKRD)Ulm, Germany; 7 Anthony Nolan Research Institute, Royal Free CampusLondon, UK; 8 UCL Cancer Institute, Royal Free CampusLondon, UK

**Keywords:** genotype, Genotype List String, human leukocyte antigen, killer-cell immunoglobulin-like receptor

## Abstract

Knowledge of an individual's human leukocyte antigen (HLA) genotype is essential for modern medical genetics, and is crucial for hematopoietic stem cell and solid-organ transplantation. However, the high levels of polymorphism known for the HLA genes make it difficult to generate an HLA genotype that unambiguously identifies the alleles that are present at a given HLA locus in an individual. For the last 20 years, the histocompatibility and immunogenetics community has recorded this HLA genotyping ambiguity using allele codes developed by the National Marrow Donor Program (NMDP). While these allele codes may have been effective for recording an HLA genotyping result when initially developed, their use today results in increased ambiguity in an HLA genotype, and they are no longer suitable in the era of rapid allele discovery and ultra-high allele polymorphism. Here, we present a text string format capable of fully representing HLA genotyping results. This Genotype List (GL) String format is an extension of a proposed standard for reporting killer-cell immunoglobulin-like receptor (KIR) genotype data that can be applied to any genetic data that use a standard nomenclature for identifying variants. The GL String format uses a hierarchical set of operators to describe the relationships between alleles, lists of possible alleles, phased alleles, genotypes, lists of possible genotypes, and multilocus unphased genotypes, without losing typing information or increasing typing ambiguity. When used in concert with appropriate tools to create, exchange, and parse these strings, we anticipate that GL Strings will replace NMDP allele codes for reporting HLA genotypes.

## Introduction

The human leukocyte antigen (HLA) genes on human chromosome 6p21 are the most polymorphic and medically relevant genes in the human genome 1–4. In April 2013, 9106 distinct nucleotide sequences at 19 HLA genes were known to encode 6617 unique HLA proteins 5. These HLA proteins are cell-surface antigens that present endogenously and exogenously derived 8–10 residue peptides for inspection by T cells, permitting the discrimination of self from nonself by the adaptive immune system 6, 7. In addition, class I HLA proteins (which present endogenous peptides) serve as ligands for killer-cell immunoglobulin-like receptors (KIR), which regulate cell killing and cytokine response as part of the innate immune system 8, 9.

The high diversity of HLA proteins is driven by their peptide binding function; each protein can present a small population of chemically similar peptides, which are bound by a peptide binding groove formed by a few dozen amino acid residues 6, 10, 11. These residues are encoded by exons 2 and 3 of the class I HLA genes (e.g. HLA-A, -B, -C, -E, -F, and -G) and by exon 2 of the class II HLA genes (DRA, DRB1, DRB3, DRB4, DRB5, DQA1, DQB1, DPA1, DPB1, DMA, DMB, DOA, and DOB) 12–14. Evolutionary mechanisms (e.g. host–pathogen coevolution) have generated a broad diversity of peptide binding groove chemistries by shuffling sets of amino-acid residues between proteins, and selection for the ability to present highly immunogenic peptides has resulted in extensive linkage disequilibrium (LD) between the individual nucleotide polymorphisms in an HLA gene 15–20. Each such set of polymorphisms in LD is known as an HLA allele, and the World Health Organization Nomenclature Committee for Factors of the HLA System (HLA Nomenclature Committee) maintains a system of allele names that describes the sequence relationships between alleles in a hierarchical fashion 14. Each allele name consists of a set of 2–4 fields that numerically identify distinct allele families, unique protein sequences, silent-substitutions, and noncoding substitutions. The name of each recognized HLA allele and its associated nucleotide and peptide sequence is curated in the IMGT/HLA Database (www.ebi.ac.uk/ipd/imgt/hla/). Since 1987 21, when only 19 distinct HLA alleles were recognized, the number of alleles has increased regularly and significantly, with growth driven by the advent of new technologies for investigating nucleotide sequence diversity 5.

Given these key roles played by HLA in the innate and adaptive immunity it is not surprising that many individual HLA alleles confer susceptibility to and protection from infectious and autoimmune diseases, pharmacological sensitivities, and cancers. More than 100 such disease-phenotype associations are known 3, and more than 1000 disease-associated HLA single-nucleotide polymorphisms (SNPs) have been identified 4. Further, the outcome of a hematopoietic stem cell (HSC) or solid-organ transplant is dependent on the degree to which the HLA alleles of patients and donors are ‘matched’; HSC transplant outcome is significantly improved for HLA-identical donor–patient pairs over ‘mismatched’ donor–patient pairs, where even a single HLA allele differs between donor and patient 22.

## HLA genotyping and ambiguity

Knowledge of an individual's HLA genotype is therefore crucial in the age of personalized genomic medicine. Ideally, knowledge of the complete nucleotide sequences of a patient's HLA genes would allow deep insight into their immune function and medical predisposition. However, the extensive polymorphism at both the nucleotide and allele levels among the HLA genes has made HLA genotyping complicated, and the ideal of certain knowledge of an individual's HLA alleles remains a distant goal. HLA nucleotide polymorphisms are often not simple biallelic SNPs; in many cases, all four nucleotide residues exist as variants of a given position, and multiple adjacent nucleotide positions (e.g. multiple codons) may be polymorphic. HLA polymorphism must often be assessed across multiple exons, and HLA genes are themselves homologous, making it difficult to assign nucleotide sequences to a particular gene.

Given these challenges, multiple polymerase chain reaction (PCR)-based techniques have been developed for HLA genotyping; the most commonly used of these are hybridization-based sequence-specific priming (SSP) and sequence-specific oligonucleotide (SSO) probe methods, and sequence-based typing (SBT) methods 23. While each method uses different approaches to assess the relevant polymorphisms necessary to identify an HLA allele, all are limited in the region of each gene that can be assessed, and in their ability to establish phase between assessed regions. These limitations can result in ambiguity—uncertainty in a genotyping result such that a method cannot identify exactly two HLA alleles for a given locus. In general, HLA genotyping results display two discrete categories of genotyping ambiguity.

*Allelic ambiguity* results when not all relevant nucleotide positions are interrogated; this type of ambiguity occurs with SSO and SSP methods when polymorphisms are located between probe or primer regions or when probes or primers cannot detect a variant, and with SBT methods when polymorphisms occur outside the region that was sequenced. For example, the HLA-A*02:03:01, HLA-A*02:253, HLA-A*02:264, and HLA-A*02:370 alleles share identical exon 2 and 3 nucleotide sequences; these alleles will constitute an ambiguous allele set when typed using an SBT method that interrogates only HLA-A exons 2 and 3. The HLA Nomenclature Committee has developed a nomenclature for describing HLA class I alleles that share identical exon 2 and 3 sequences, and HLA class II alleles that share identical exon 2 sequences. All such alleles are assigned to a ‘G group’ named using the first three fields of the lowest-numbered allele in that ambiguous allele set, followed by the letter G 14. Thus the HLA-A*02:03:01, HLA-A*02:253, HLA-A*02:264, and HLA-A*02:370 alleles are all part of the HLA-A*02:03:01G group. This G group nomenclature is useful for representing ambiguous alleles generated via SBT methods, but SSO methods may not have the capacity to assess all polymorphisms in the relevant exons, and may therefore generate even more ambiguous results.

*Genotypic ambiguity* results when chromosomal phase cannot be established between polymorphisms; this type of ambiguity also occurs with SSP, SSO, and SBT methods. For example, the ‘HLA-A*01:01:03 and HLA-A*02:01:04’ and ‘HLA-A*01:01:01 and HLA-A*02:01:18’ genotypes are consistent with the same set of diploid exon 2 and 3 nucleotide sequences, and will constitute an ambiguous genotype combination when typed using an SBT method that does not establish phase between HLA-A exons 2 and 3 24.

The extent of allelic and genotypic ambiguity can be large in some common HLA genotypes. For example, in release 3.9.0 of the IMGT/HLA Database 5, the ambiguous genotype combinations that correspond to the four exon 2 and 3 nucleotide sequences represented by the HLA-A*02:01:01G and HLA-A*03:01:01G G groups include 555 genotypes when these G groups are expanded to their constituent alleles. The number of genotype combinations in this case can be considerably higher when an SSOP or SSP method is used rather than an SBT method.

Finally, the regular identification of new HLA alleles has made the consistent management of HLA genotype data challenging. An HLA genotyping result that may be unambiguous at one point in time may become ambiguous at a later date, when a new nucleotide variation is reported that was not excluded at the time of the original typing.

## Recording genotyping ambiguity

Historically, there has been a lack of consensus with respect to the recording of allelic and genotypic ambiguities. The HLA Nomenclature Committee recommends the use of the forward slash (/) and comma (,) as operators for reporting allele ambiguity (e.g. the above-mentioned ambiguous allele set can be recorded as HLA-A*02:03:01/02:253/02:264/02:370, which is generally referred to as an ‘allele string’) and distinguishing the diploid alleles at a locus (e.g. the above-mentioned genotypes can be recorded as HLA-A*01:01:03, 02:01:04 and HLA-A*01:01:01, 02:01:18), but there is no standard method for recording ambiguous genotype combinations. In particular when ambiguity is extensive, laboratories often only report the lowest-numbered allele pair; this is a dangerous simplification that contradicts most standards.

The most commonly used approach for reporting and transmitting ambiguous HLA genotype data has been to use the allele code system developed by the NMDP. This system replaces the 2nd–4th fields of an allele name with a 2–5 letter code that represents an allelic ambiguity string. For example, the ambiguous HLA-A*01:01/01:02, HLA-A*02:01/02:24/02:101 genotype is coded as HLA-A*01:AB, HLA-A*02:CVEG. When NMDP allele codes were first introduced in the 1990s, only a few hundred HLA alleles had been identified and it was assumed that only a small number of alleles remained to be identified. Since then, the number of allele codes has grown extremely large in response to the growth in number of HLA alleles. As of March 2013, 200,047 distinct allele codes have been assigned. Applied to multiple allele-families across the HLA loci, several million unique allele codes can be generated.

While use of allele codes is preferable to the simple truncation of the allele string, in that it allows more complete recording and transmission of a genotyping result, the NMDP allele code system remains an imperfect method of recording and transmitting modern HLA genotype data for the reasons that follow.

## Limitations of allele codes

### Inability to encode genotype ambiguity

The NMDP allele code system cannot encode genotypic ambiguity. Genotypic ambiguity must be ‘compressed’ into allelic ambiguity before a typing can be encoded. Therefore, any phase information in the genotyping result cannot be represented in the allele code and is lost in the encoding process. For example, a typing result of two possible genotypes of HLA-A*02:01, HLA-A*11:08 or HLA-A*02:02, HLA-A*11:20 will be reported as HLA-A*02:AB, HLA-A*11:HNF, which expands into the following four possible genotypes: HLA-A*02:01, HLA-A*11:08 or HLA-A*02:02, HLA-A*11:20 or HLA-A*02:01, HLA-A*11:20 or HLA-A*02:02, HLA-A*11:08.

When genotypic ambiguities are converted to allele codes, new genotypes not included in the original genotyping result are introduced, and phase information for that locus is completely lost.

### Outmoded assumptions about HLA polymorphism

The NMDP allele code system generally assumes that most ambiguity will pertain to the 2nd field of an allele name. Ambiguities that pertain to the 3rd and 4th fields of allele names cannot be recorded, because allele codes only represent amino acid sequences. For example, the HLA-A*02:01:05/02:02:02 ambiguity is shortened to HLA-A*02:01/02:02 prior to encoding, and is coded as HLA-A*02:AB. A different allelic ambiguity (e.g. HLA-A*02:01:04/02:02:01) is also encoded to HLA-A*02:AB. An ambiguity derived from synonymous substitutions, such as HLA-A*02:01:06/02:01:07, cannot be encoded. Therefore, when genotyping results are converted to allele codes, new ambiguity is introduced and information in the 3rd and 4th fields of allele names is lost.

In addition, the NMDP allele code system generally assumes that there will be no ambiguity in the 1st field of allele names. With the exception of the DPB1 locus, the 1st field of an allele name represents a specific ‘allele family’ at a given locus. These allele families have historically corresponded to specific immunogenic peptide domains, and were originally defined via serological typing. However, as the number of known alleles has increased, alleles that cannot easily be assigned to a specific allele family on the basis of nucleotide sequence have been identified. As a consequence of these serologically uncertain alleles and the patchwork structure of the HLA polymorphism, an increasing number of genotyping results now include ambiguities involving the 1st field. This leads to a growing numbers of allele codes crossing those generic groups.

NMDP allele codes cannot be generally applied to alleles that are in different allele families. For example, an ambiguous typing result of HLA-A*02:03:01/02:253/23:17 cannot be converted to an allele code. Although some specific cross-family allele codes have been created (e.g. the HLA-DRB1*13:DJ code represents the HLA-DRB1*13:01/13:02/13:04/13:05/13:06/13:07/14:09 allelic ambiguity), allele codes that specifically incorporate ambiguities in the first field of allele names are primarily used only for the DPB1 locus (for which the concept of an allele family does not apply). Because the NMDP allele code system cannot easily accommodate ambiguities in the 1st field, additional HLA typing is often used to exclude these ambiguities, increasing the cost and time required to report a genotype.

### Allele code management bottlenecks

New NMDP allele codes are generated and managed in a nonautomated fashion. With each release of an update from the HLA Nomenclature Committee, the overall number of alleles increases. As genotyping efforts are extended to exons that have not previously been examined, new polymorphisms are found in what were thought to be well-characterized alleles. As a result, ambiguity increases with each new release of the IMGT/HLA Database, and hence a previously unambiguous genotype can later become ambiguous. If an NMDP allele code corresponding to an ambiguity does not exist, or has not been activated for use at a particular locus, the creation of a new code, or the activation of an existing code at a new locus, must be requested. This constitutes a rate-limiting step so far as the efficient recording and transmission of HLA genotype data goes.

These issues have resulted in recommendations that NMDP allele codes not be used in HLA reports 24–26 but so far no specific alternatives have been provided. Other machine-readable formats have been developed but these require specific programming skill to use 27. Here, we describe Genotype List (GL) Strings, a machine-readable and human intelligible syntax for reporting HLA genotype results that allows the accurate recording of allele and genotype ambiguity, as well as the integration HLA genotyping results with data from other genetic systems.

## Methods and results

**Figure 1:**
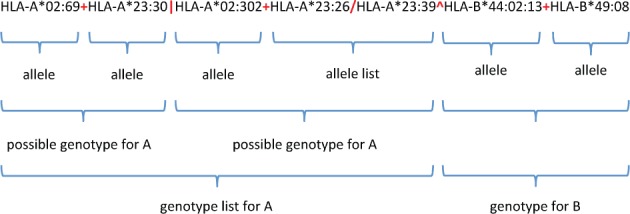
Genotype List (GL) String representation of a multilocus unphased genotype. A GL String representing HLA-A genotype (A*02:69 and A*23:30, or A*02:302 and, either A*23:26 or A*23:39) and HLA-B genotype (B*44:02:13 and B*49:08) for a single individual is shown. GL String delimiters are parsed hierarchically starting from the locus delimiter (^), proceeding to the genotype delimiter (|), then the chromosome delimiter (+), and ending with the allele delimiter (/).

A GL String is a collection of alleles parsed with character delimiters that organize the alleles in terms of loci, alleles, lists of possible alleles, phased genes, genotypes, and lists of possible genotypes. These delimiters, their meaning and the precedence in which they must be applied are presented in Table 1. An example GL String is illustrated in Figure 1. A reduced set of these delimiters was previously proposed for reporting KIR genotype data 28. The delimiters are described in greater detail below, in order of decreasing precedence.

**Table 1:** Genotype List String data format definition and precedence

Precedence^a^	Delimiter	Description	Example
5	/	Allele ambiguity	HLA-A^*^02:01/HLA-A^*^02:02
4	∼	Phased genes	HLA-DRB3^*^01:01∼HLA-DRB1^*^03:01
3	+	Copies of genes	HLA-A^*^02:01/HLA-A^*^02:02+HLA-A^*^03:01
2	|	Genotype ambiguity	HLA-A^*^02:01/HLA-A^*^02:02+HLA-A^*^03:01|HLA-A^*^02:07+HLA-A^*^03:06
1	^	Genes	HLA-A^*^02:01/HLA-A^*^02:02+HLA-A^*^03:01|HLA-A^*^02:07+HLA-A^*^03:06^ HLA-B^*^08:01+HLA-B^*^44:02/HLA-B^*^44:03

a Precedence is evaluated in the numerical order shown.

### Ambiguous alleles

The forward slash character (/) is used to separate possible alleles in a list. For example, HLA-A*02:01/HLA-A*02:02 denotes two possible alleles. This delimiter has been in common use among HLA researchers and has been previously recommended by the HLA Nomenclature Committee for reporting an ambiguous string of alleles 14.

### Phased genes

The tilde character (∼) is used to separate alleles that are found on the same chromosome and is used to group alleles within the same haplotype (*cis*). For example, HLA-DRB3*01:01∼HLA-DRB1*03:01 describes two HLA-DRB alleles that have been identified as being on the same chromosome.

### Copies of genes

The plus character (+) separates alleles that are detected, but not identified as being on the same chromosome. In HLA genotypes, this character is most often used to denote genotypes at a locus, with the alleles found on different chromosomes (*trans*). However, it can also be used to denote copies of genes where chromosomal phase is unknown. For example, it has been used to represent more than two copies of a gene in reporting KIR genotype data 28.

### Ambiguous genotypes

The pipe character (|) is used to separate possible genotypes in a list. For example, HLA-A*02:02+HLA-A*03:01|HLA-A*02:07+HLA-A*03:06 denotes two possible genotypes for HLA-A.

### Genes/loci

The caret character (^) is used to separate loci and is used to describe multilocus unphased genotypes. For example, HLA-A*02:01+HLA-A*03:01^HLA-B*08:01+HLA-B*44:02 describes two genotypes, one each for the HLA-A and HLA-B loci.

Other than these five delimiters (/, ∼, +, |, and ^) and appropriate allele identifiers, no additional characters should be included in GL Strings. For example, white spaces and tabs must be excluded from GL Strings. We recommend that HLA allele names in GL Strings be strictly consistent with the IMGT/HLA Database and always be prefaced with ‘HLA-’ to explicitly identify HLA data, and that they always include the full locus name (e.g. A, DRB1, etc.), the asterisk (*) separator, and the allele designation. For example, the following ambiguous HLA-A allele pair should always be written as HLA-A*01:01:01:01/HLA-A*02:01:01:02L, and never as HLA-A*01:01:01:01/02:01:01:02L.

The order of delimited elements in a GL String does not provide any additional meaning for parsing that string. For example, the order of slash-delimited alleles or pipe-delimited genotypes does not indicate any greater likelihood of one allele or one genotype over another. Similarly, the order of loci in a GL String does not need to correspond to the relative chromosomal position of those loci. Neither is there any meaning from order of loci in a GL String with regard to relative chromosomal position.

The pertinent IMGT/HLA Database release version of a given allele is not included in a GL String. It is the responsibility of the creator of the string or the tool generating the string to convey the IMGT/HLA Database version and associated metadata to the recipient of the GL String.

Genotype data for any genetic systems that use a standard nomenclature for identifying polymorphisms can be represented with GL Strings so long as that nomenclature of the genetic system does not make use of the GL String delimiters. For example, genotype data for multiple KIR loci for a single individual could be represented in a single GL String, as: KIR2DL1*001 + KIR2DL1*001^KIR2DL2*0010101 + KIR2DL2*0030101^KIR2DL5A*0010101 + KIR2DL5A*014^KIR2DL5B*020101 + KIR2DL5B*003^KIR2DS3*00101 + KIR2DS3*0020101^KIR2DS4*0010101 + KIR2DS4*002|KIR2DS4*0040101 + KIR2DS4*0060102^KIR2DS5*003/KIR2DS5*004/KIR2DS5*005 + KIR2DS*001.

## Discussion

We have developed a string format that can fully describe HLA genotyping results. By applying character delimiters with defined precedence, GL Strings can be used to record allele and genotype ambiguity in a standard manner that does not increase ambiguity or lose information. The adoption of this format as a standard means for recording HLA genotype data could have widespread ramifications for basic and clinical research in the fields of histocompatibility and immunogenetics. A key obstacle to consistency and reproducibility of immunogenomic studies has been the inability to determine the extent to which genotype data generated by different research groups, using different methodologies and platforms, and at different times represent equivalent results 24. The ability to store and exchange HLA genotyping results that accurately represent allelic and genotypic ambiguity will potentially overcome this obstacle, facilitating the synthesis of data across platforms, research groups, and nomenclature epochs.

The GL String format can also be used for other genetic systems with defined nomenclatures (e.g. KIR) as long as those nomenclatures do not use the GL String character delimiters. This flexibility allows data for multiple genetic systems to be associated in ways that are not currently possible. For example, it is possible to incorporate HLA and KIR data for a given individual in the same GL String. However, we discourage the combination of genotype data using multiple nomenclatures (or multiple versions of the same nomenclature) in a single GL String, as the GL String format does not associate specific nomenclatures with the individual elements of a GL String.

### Other formats

The need to accurately record allele variation is not unique to HLA research. An understanding of sequence variation is foundational to the promise of personalized genomics, and several different genomic data formats have been described. Reese et al. have described a Genome Variation Format (GVF) that is a type of Generic Feature Format (GFF) to be used with the 10Gen dataset 29. For the 1000 Genomes project, a Variant Call Format (VCF) 30 consisting of a text file containing metadata lines, a header line, and data lines containing positional information has been developed. These genomic formats are not applicable for nomenclature systems, as they represent variation presented in the context of a reference genome.

### Managing and using GL Strings

An important goal in the development of GL Strings was to separate the encoding of genotype data from the management and presentation of those data. Despite their shortcomings, NMDP allele codes have been popular because they compress information into a small amount of printable real estate and can be easily exchanged using paper records. However, as discussed above this compression greatly reduces the utility of HLA genotype data, and the management and maintenance of allele codes is time-consuming.

As with allele codes, GL Strings have the potential to become quite numerous and difficult to read. However, they are easily generated and parsed by computers and the work of creating and displaying them should be left to machines. The remaining challenge is one of exchanging the strings easily.

While it is possible to develop something akin to the allele code system for the representation of unique GL Strings, a more desirable solution would be to register each string with a service that returns a unique Uniform Resource Identifier (URI) 31 that can be easily dereferenced and with the ability to return the string in multiple formats, as required by the application requesting the information. Such a service is currently under development. By eliminating the manual steps required to curate allele codes, and by enlisting computational resources for managing GL Strings, the management and process issues associated with the NMDP allele code system become moot.
